# Therapeutic effects of probiotics on symptoms of depression in children and adolescents: a systematic review and meta-analysis

**DOI:** 10.1186/s13052-024-01807-6

**Published:** 2024-11-07

**Authors:** Chia-Min Chen, Shun-Chin Liang, Cheuk-Kwan Sun, Yu-Shian Cheng, Yen-Hsiang Tang, Cheng Liu, Kuo-Chuan Hung

**Affiliations:** 1 Department of Natural Biotechnology, Nanhua University, Chiayi, Taiwan; 2 Department of Psychiatry, Jianan Psychiatric Center, Ministry of Health and Welfare, Tainan, Taiwan; 3 Department of Center for General Education, University of Kun Shan, Tainan, Taiwan; 4 Department of Optometry, University of Chung Hwa of Medical Technology, Tainan, Taiwan; 5 Department of Emergency Medicine, E-Da Dachang Hospital, I-Shou University, Kaohsiung City, Taiwan; 6 School of Medicine, College of Medicine, I-Shou University, Kaohsiung City, Taiwan; 7 Department of Psychiatry, Tsyr-Huey Mental Hospital, Kaohsiung Jen-Ai’s Home, Kaohsiung City, Taiwan; 8 Department of Critical Care Medicine, MacKay Memorial Hospital, Taipei, Taiwan; 9 Department of Medicine, MacKay Medical College, New Taipei City, Taiwan; 10 Department of Physical Education, Huazhong University of Science and Technology, Wuhan, China; 11 Department of Anesthesiology, Chi Mei Medical Center, Chi Mei Medical Center, No.901, ChungHwa Road, YungKung Dist, Tainan, 71004 Taiwan

**Keywords:** Probiotics, Depression, Meta-analysis

## Abstract

This meta-analysis aimed at investigating the therapeutic effects of probiotics against the symptoms of depression in children and adolescents as well as to identify the potential confounders. Following PRISMA guidelines, major databases were searched for randomized controlled trials focusing on effects of probiotics against the symptoms of depression in children and adolescents to analyze the effect size (ES) for primary outcomes (i.e., improvement in depressive symptoms) expressed as standardized mean difference (SMD) and odds ratios (ORs) for continuous and categorical variables, respectively, with 95% confidence interval (CI). Meta-analysis of five studies (692 participants, mean age = 7.33 years, treatment duration 8–104 weeks) demonstrated no significant improvement in depressive symptoms in subjects receiving probiotics (SMD = 0.04, 95% CI: -0.33 to 0.41,* p* = 0.84, five studies, 692 participants). Subgroup analysis also showed no significant improvement associated with probiotic use relative to controls in the subgroup of studies focusing on individuals diagnosed with neurodevelopmental disorders (SMD = -0.11, 95% CI: -0.73 to 0.51,* p* = 0.72, three studies, 452 participants) and that recruiting the general population (SMD = 0.24, 95% CI: -0.43 to 0.91, *p* = 0.48, two studies, 240 participants). However, high levels of heterogeneity were found in both our primary results (I2 = 77%, *p* = 0.001) and subgroup analyses for those with neurodevelopmental disorders (I2 = 84%,* p* = 0.002) and the general population (I2 = 79%, *p* = 0.03). The results did not support the use of probiotics for relieving depressive symptoms compared with controls in children and adolescents diagnosed with neurodevelopmental disorders or in the general population. Nevertheless, given the high level of heterogeneity across the included trials and a lack of studies focusing on those with diagnoses of anxiety or depression in the current meta-analysis, further large-scale clinical investigations are required to elucidate the therapeutic potential of probiotics against depressive symptoms in these populations, especially in those diagnosed with neurodevelopmental disorders or depression.

**Supplementary Information:**

The online version contains supplementary material available at 10.1186/s13052-024-01807-6.

## Introduction

Probiotics have been used for treating a variety of somatic problems [1], and several neuropsychiatric disorders [2–8]. Indeed, a recent systematic review reported potential beneficial effects of probiotics against not only certain allergic problems such as atopic/eczema but also a variety of inflammatory diseases including inflammatory bowel diseases, rheumatoid arthritis, and bronchial asthma through modulation of T-cells and cytokines [9]. As for neuropsychiatric problems, their possible modulating effects can be attributed to the crosstalk between enteral microbiome and the central nervous system (CNS) via the neuroendocrine network of the gut-brain-axis (GBA) [10], as well as the anti-inflammatory and antioxidant properties of some probiotics [11, 12].

However, although probiotics-associated neurotransmitter modulation via GBA may be beneficial to mood regulation and cognitive function in general [13], their efficacy in relieving neuropsychiatric symptoms may vary with disease entities and age [2–8]. For instance, despite the report of some positive probiotics effects in the adult population on depression [5] and cognitive functions [8], most meta-analyses focusing on children and adolescents failed to show benefits of probiotics in the treatment of attention [3], cognitive function [4] and symptoms of autism spectrum disorders (ASD) [6]. Therefore, although a previous meta-analysis seemed to support the therapeutic use of probiotics for depressive symptoms in the adult population [5], the finding may not be extrapolated to children and adolescents.

On the other hand, although most previous meta-analyses demonstrated no significant therapeutic effects of probiotic use against neurodevelopmental problems in children and adolescents, benefits were reported under certain conditions such as a longer duration of probiotic administration [4], or the use of multiple-strain regimens [6] in the treatment of cognitive functions [4] or symptoms of ASD in children and adolescents. Another factor that may affect the treatment efficacy of probiotics may be the nature of neurodevelopmental disorders. For example, previous experimental studies using a murine model of ASD showed an increased intestinal permeability [14], which was alleviated after oral administration of probiotics containing *B. fragilis* [15]. This finding was clinically supported by that of a prior meta-analysis targeting children and adolescents that showed the efficacy of probiotic blends against the overall behavioral symptoms of ASD [6], but not the symptoms of attention deficit/hyperactive disorders (ADHD) [3]. Moreover, another meta-analysis on adults reported a significant improvement in depressive symptoms compared with controls only in those diagnosed with depressive disorders and not in those only under stress but without such diagnoses [5]. Taken together, certain factors including age, treatment duration, number of probiotic strains, and disease entity may influence the therapeutic effectiveness of probiotics.

Therefore, the aims of the current meta-analysis were to investigate the therapeutic effects of probiotics against the symptoms of depression in children and adolescents as well as to elucidate the significance of potential factors that may affect treatment efficacy.

## Methods

### Protocol and registration

The current meta-analytic investigation, which was registered in the PROSPERO international prospective register of systematic reviews (CRD42024532877), was conducted in compliance with the Preferred Reporting Items for Systematic Reviews and Meta-Analyses (PRISMA) guidelines [16].

### Search strategy and selection criteria

Based on the keywords pertinent to the current study, electronic databases including PubMed, Embase, ScienceDirect, Cochrane CENTRAL, and ClinicalTrials.gov were searched from inception to May 2, 2024 (eTable 1) for randomized controlled trials (RCTs) that studied the therapeutic efficacy of probiotics against the symptoms of depression regardless of language and country of publication. To avoid missing eligible trials, the reference lists of the initially retrieved articles were examined in detail. Study eligibility was based on the criteria of PICO (i.e., population, intervention, comparator, and outcome): (1) Population: children or adolescents aged 18 or below, (2) Intervention: probiotics or products containing probiotics, (3) Comparator: non-probiotic interventions or placebo, and (4) Outcome: severity of the symptoms of depression. Studies that (1) did not include probiotics as interventions, (2) were not RCTs, or (3) failed to provide data on symptoms of depression as their outcome measures were excluded.

### Data extraction and quality assessment

Two authors (PW Huang and SC Liang) independently examined the titles and abstracts of the retrieved articles that were identified based on the preset keywords and search strategies (eTable 1). The two authors also extracted data on study characteristics and outcomes. A third author (CK Sun) acted as arbitrator in case of disagreements regarding study or data eligibility. The corresponding authors of papers with missing data were contacted through emails in an attempt to retrieve the necessary information. The quality of the included studies and their evidence level of the derived outcomes were determined with Cochrane's “risk of bias” assessment tool [17], and the Grading of Recommendations Assessment, Development, and Evaluation (GRADE) [18] respectively. All disagreements were resolved through discussion.

### Data synthesis and analysis

The primary outcomes of the current study were changes in the symptoms of depression assessed with standardized evaluation tools [e.g., the Children's Depression Inventory (CDI), the Center for Epidemiologic Studies Depression Scale (CES)] or other validated rating scales. The secondary outcomes included the participants withdrawn from a trial (i.e., acceptability) and those withdrawn due to adverse events (i.e., tolerability) after receiving probiotic treatments in comparison with control groups. Effect size (ES), which was used to quantify treatment outcomes, was shown as odds ratios (ORs) and standardized mean differences (SMDs) for categorical and continuous parameters, respectively, with 95% confidence intervals (CIs). SMD was used because the outcomes measures included a variety of scales for rating depressive symptoms. Review Manager 5 (RevMan 5.4; Copenhagen: The Nordic Cochrane Center, The Cochrane Collaboration, 2014) was adopted for all statistical analyses using the Mantel–Haenszel and generic inverse-variance methods for assessing the significance of difference for categorical and continuous data, respectively. Subgroup analyses were conducted to investigate the potential influence of different neurodevelopmental disorders (i.e., ASD and ADHD), the number of microbiome strains in a probiotic regimen, as well as treatment duration on the therapeutic outcome (i.e., changes in the symptoms of depression). We examined the robustness of evidence by appraising the impact of each of the included trials on the overall outcome using leave-one-out sensitivity tests. In addition, we evaluated the degree of heterogeneity across the studies with I-squared tests. Furthermore, we inspected a funnel plot to discern potential publication bias. Statistical significance for all outcomes was defined as a probability value, *p*, less than 0.05.

## Results

### Eligible studies and characteristics

**Fig. 1:**
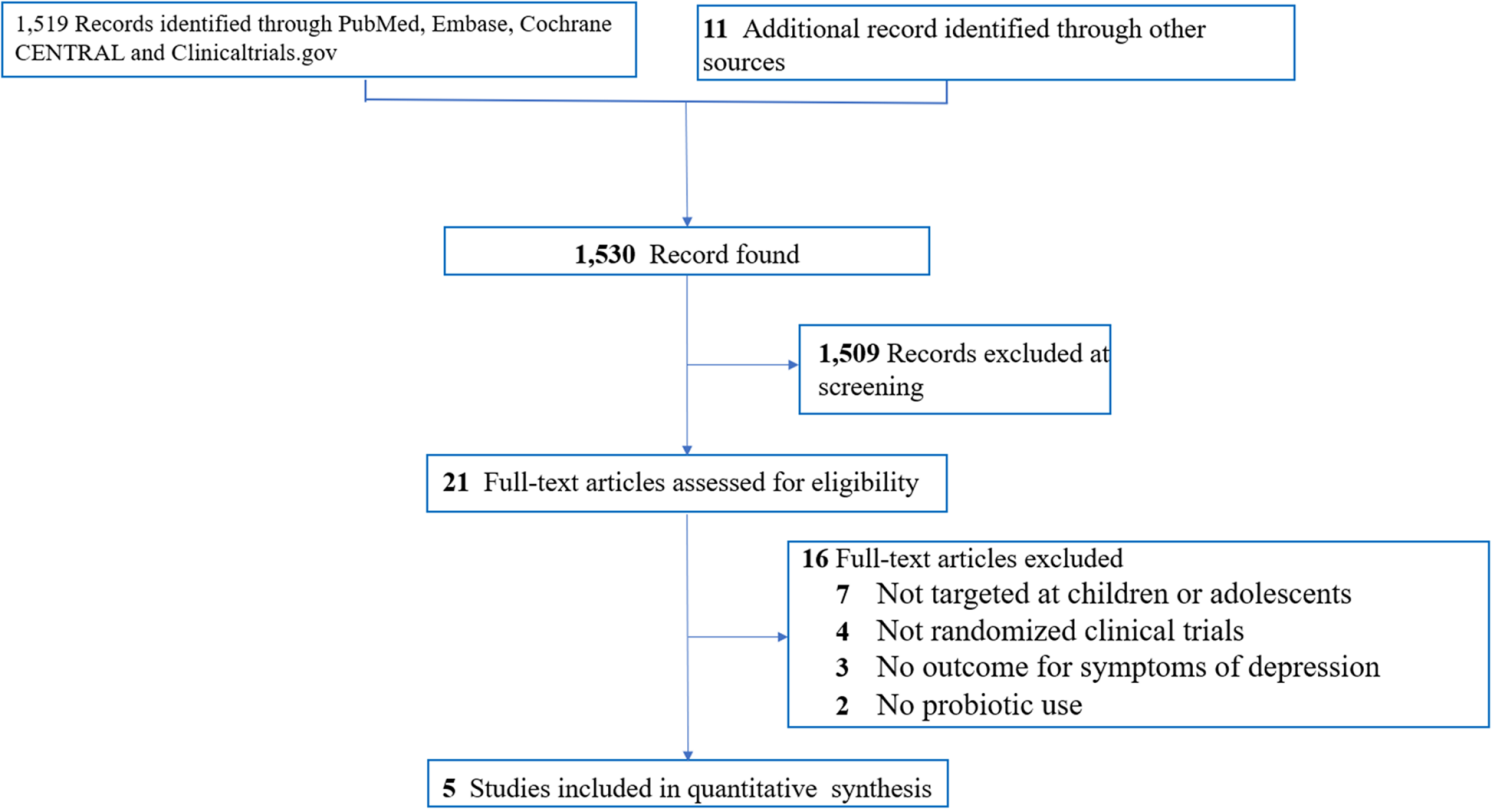
PRISMA diagram of identifying eligible studies

In compliance with the PRISMA statement [16], 1530 articles were initially retrieved from the electronic databases based on the selected keywords and search strategies (eTable 1). 1530 articles were initially retrieved from the electronic databases based on the selected keywords and search strategies (eTable 1). After title and abstract screening, 1,509 articles comprising studies that were not RCTs, those included adults in their analysis, those in which depression was not an outcome measure, and those that were duplicates from different databases were excluded. Finally, 21 studies were deemed suitable for full-text scrutiny that identified five eligible RCTs including 692 participants [19–23] (Fig. 1) (eTable 2). Data from the eligible studies were extracted on May 02, 2024. All five trials recruited children or adolescents. Because two studies that enrolled infants and children from the general population did not provide information about age [21, 23], the mean age of the participants was 7.33 based on the other three trials (Table 1). Regarding the diagnosis, two studies enrolled infants and children without a disease diagnosis [21, 23], one focused on participants diagnosed with ASD [19], one targeted those diagnosed with ADHD [20], and one investigated those diagnosed with Tourette’s syndrome [22]. In terms of the number of probiotic strains, four trials focused on single-strain probiotics [19, 21–23] and the other adopted multiple-strain regimens [20]. The median duration of treatment was eight weeks (range: 8–104 weeks). In respect of study design, all studies adopted a parallel design [19–23]. The countries of origin of the included studies were Australia and New Zealand [21], Taiwan [19, 22], Indonesia [23] and Iran [20].

**Table 1:** Summary of characteristics of studies in the current meta-analysis

Study (year)	Diagnosis (Criteria)	Design	Comparison	N	Duration (weeks) /FU time	Outcome	Psychotropic medications	Mean age (years)	Female (%)	Country
Liu [8]	ASD (DSM-5)	RCT	Probiotics: single strain (L. plantarum)	41	8 /post-treamtent	CBCL anxious/ depressed	Allow only psychostimulant	4.82 (2.5–7)	12.2	Taiwan
			Placebo	41						
Rianda [23]	Children (1-6 yrs) in general population	RCT	RC + Probiotics: single strains ( L. reuteri)	126	24 /10 ys later	CDI	N/A	15.31 (10-18)	53.0%	Indonesia
			RC + Probiotics: single strains (L. casei)	120						
			RC	124						
Sepehrmanesh [20]	ADHD (DSM‑IV‑TR)	RCT	Probiotics: multiple strains^a^ + MPH	20	8 /post-treamtent	CDI	100% MPH	9.1 (8-12)	20.6	Iran
			Placebo + MPH	20						
Wu [22]	Tourette syndrome (DSM-5)	RCT	Probiotics: single strain (L. plantarum)	28	8 /post-treamtent	CDI	Allowed	9.86 (5-18)	15.79	Taiwan
			Placebo	29						
Slykerman [21]	Infant in general population	RCT	Probiotics: single strains (L. rhamnosus)	104	104 /4 ys later	CES-DC	N/A	Treatment started at 35 gestation	N/A	New Zealand
			Probiotics: single strains (B. animalis)	97						
			Placebo	97						

*ADHD* Attention-deficit/hyperactivity disorder, *ASD* Autism spectrum disorder, *B*. Bifidobacterium, *CBCL* Child Behavior Checklist, *CDI* Children's Depression Inventory, *CES-DC* Center for Epidemiological Studies Depression Scale for Children, *DSM-5* Diagnostic and statistical manual of mental disorders fifth edition, *DSM-IV-TR* Diagnostic and Statistical Manual of Mental Disorders, fourth edition, text revision, *FU* Follow up, *MPH* Methylphenidate, *N* Number, *N/A* Not available, *L.* Lactobacillus, *RC* Regular calcium supplementation, *RCT* Randomized controlled trial, *yrs* years

Multiple strains^a^: including Lactobacillus reuteri, Lactobacillus acidophilus, Lactobacillus fermentum, and Bifidobacterium bifidum

### Risk of bias appraisal

**Fig. 2:**
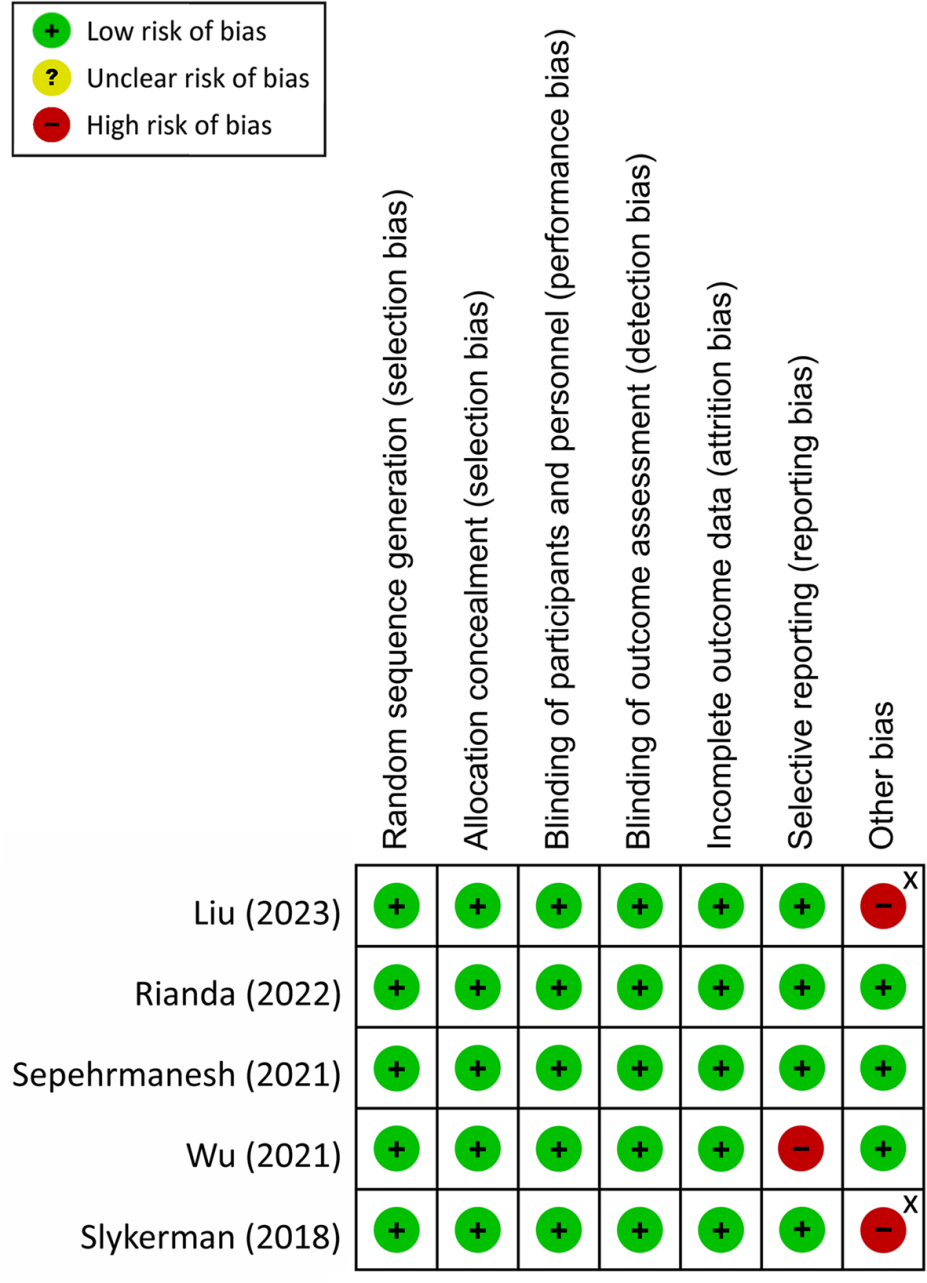
Risk of bias for eligible studies. ^X^ Study that received financial support from private companies

The risk of bias of individual studies appraised with the Cochrane Collaboration’s tool showed low risks of allocation concealment and randomization sequence biases in most studies (Fig. 2). Moreover, detection and performance biases were considered low in all trials due to their double-blind designs. Reporting bias was deemed high in one trial in which the symptoms of depression were not the primary outcome of interest [22] (Fig. 2). A high risk of attrition bias was given to two studies [23, 24] taking into account their high rates of dropouts (Fig. 2). Another two trials were rated as having a high risk of other bias because of sponsorship from private companies [19, 21] (Fig. 2).

### Primary outcome

**Fig. 3:**
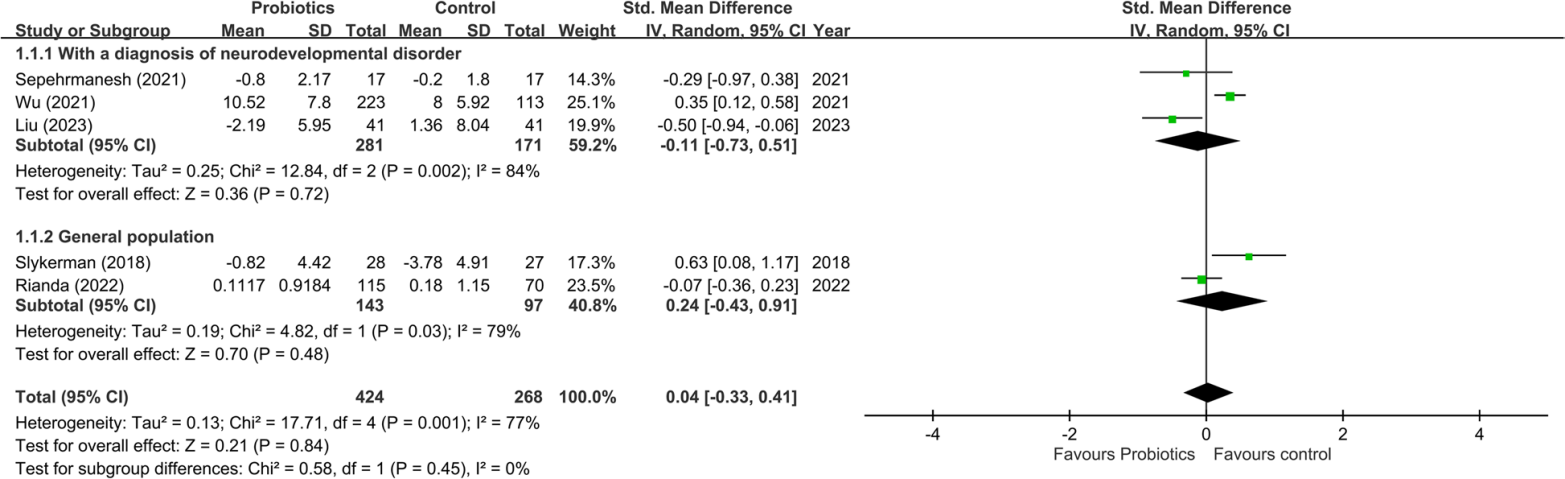
Forest plot of effect size for comparing the difference in improvement of symptoms of depression between probiotics and control groups with subgroups of those who was diagnosed with neurodevelopmental disorders or those without diagnosis of neurodevelopmental disorders. CI: confidence interval; Std: standardized; SE: standard error

Compared with the controls, the present study found no significant improvement in the symptoms of depression in subjects receiving probiotics (SMD = 0.04, 95% CI: -0.33 to 0.41, *p* = 0.84, five studies, 692 participants) (Fig. 3). Our leave-one-out sensitivity analysis showed no significant impact of any single trial on the overall outcome. A funnel plot inspection also demonstrated no asymmetry for the primary outcome (eFigure 1). On the other hand, an overview of the five studies revealed significant heterogeneity (*I*^22^ = 77% and *p* = 0.001). To elucidate the sources of heterogeneity, we conducted subgroup analyses targeting potential confounders. Our results showed no significant improvement in depressive symptoms associated with probiotic use relative to control in the subgroup of studies focusing on individuals diagnosed with neurodevelopmental disorders (SMD = -0.11, 95% CI: -0.73 to 0.51, *p* = 0.72, three studies, 452 participants) and that recruiting the general population (SMD = 0.24, 95% CI: -0.43 to 0.91, *p* = 0.48, two studies, 240 participants) (Fig. 3). Significant heterogeneity was still found in both subgroups (*I*^22^ = 84%, *p* = 0.002 and *I*^22^ = 79%, *p* = 0.03, respectively) (Fig. 3). We were unable to conduct subgroup analyses to assess the impact of the number of probiotic strains, age of participants, and treatment duration on the therapeutic effect of probiotics against the symptoms of depression due to the limited number of available RCTs.

### Secondary outcomes

No significant difference was noted in the overall number of dropouts between the probiotics and control groups (OR = 1.07, 95% CI: 0.76 to 1.50, *p* = 0.70, five studies, 1004 participants) (eFigure 2). Moreover, the results of our secondary outcomes showed no significant heterogeneity, inconsistency on sensitivity analysis, or funnel plot asymmetry suggestive of publication bias (eFigure 3). Nevertheless, dropouts due to adverse events could not be analyzed due to data unavailability in most studies.

### Certainty of evidence

The certainty of evidence of the study outcomes based on the GRADE guidelines is summarized in eTable 3. The evidence related to our primary outcome (i.e., improvements in the symptoms of depression) was downgraded to very low due to serious imprecision from the limited number of eligible studies combined with significant inconsistency arising from notable heterogeneity across the studies. Besides, the certainty of evidence on the number of dropouts was downgraded to low considering the small sample sizes.

## Discussion

To the best of our knowledge, our study was the first meta-analysis to investigate the therapeutic effects of probiotics against the symptoms of depression in children and adolescents. Nevertheless, we found only a limited number of RCTs focusing on this issue. Overall, our results based on five trials with 692 participants showed no significant difference in the degree of improvement in depressive symptoms between those treated with probiotics and the controls. Discretion is needed when interpreting our findings taking into account the significant heterogeneity from the diversity of participants ranging from normal individuals [21, 23] to those with different neurodevelopmental disorders [19, 20, 22]. Such heterogeneity, together with the limited number of eligible RCTs as well as an absence of study targeting those diagnosed with depressive disorders, precluded a robust conclusion based on the evidence of the current study to rule out the therapeutic effect of probiotics against depressive symptoms in children and adolescents. Further studies focusing on participants with neurodevelopmental disorders, especially those diagnosed with depression, are warranted to elucidate this issue.

The potential treatment benefits of probiotics are primarily attributed to their roles as immunoregulators and anti-inflammatory agents that are effective not only for neuropsychiatric problems [2–8], but also against diseases such as atopic/eczema dermatitis syndrome and food allergy, as well as inflammatory-based diseases [9]. Indeed, a previous meta-analysis recruiting adults only with stress and those diagnosed with depression or anxiety that reported an overall improvement in depressive symptoms [5]. However, our findings demonstrated no significant therapeutic effect of probiotics against depressive symptoms in children and adolescents. Despite the discrepancy in overall outcome, our result that demonstrated no notable alleviation in depressive symptoms in the normal population compared with the controls was consistent with that of subgroup analysis of that meta-analytic study that showed no significant improvement in depressive symptoms in stressful individuals without a diagnosis of depression or anxiety [5]. Therefore, the results of both our study and that meta-analysis [5] did not support the use of probiotics for treating mood symptoms in the general population either in adults or in children/adolescents. Nevertheless, because our subgroup analysis on the normal population only included two studies with 240 participants [21, 23], our finding needs to be interpreted with caution.

When focusing on those with neurodevelopmental disorders, our results were in favor of probiotics despite the lack of statistical significance. However, significant heterogeneity remained in our analysis even after exclusion of studies focusing on the general population. In addition to the known anti-inflammatory effect of probiotics [11, 12], other probiotics-associated therapeutic benefits specific to different neurodevelopmental disorders have been reported. For instance, using a mouse model of ASD, a prior study revealed an abnormal increase in intestinal mucosal permeability that resulted in a systemic elevation in inflammatory substances that in turn aggravated the ASD-like behavior [14]. In addition, another study based on the same murine ASD model showed a correction of the leaky gut epithelium after administration of *Bacteroides fragilis* (a probiotic) [15]. Compatible with these findings, a prior meta-analysis demonstrated that oral administration of probiotics blends was associated with an alleviation of the overall behavioral symptoms of ASD [6], while another meta-analytic study showed no significant therapeutic effect of probiotics against the symptoms of ADHD [3]. Therefore, these findings may suggest specific roles of probiotics in different neurodevelopmental disorders. Indeed, in current meta-analysis, only one study showed a significantly superior treatment effect of probiotics to that of the controls on the symptoms of depression in those diagnosed with ASD [19], while two other studies that focused on ADHD [20] and Tourette’s syndrome [22], respectively, failed to show a significant difference in therapeutic effects against depressive symptoms between the probiotics and control groups. Therefore, despite our overall finding of a non-significant therapeutic effect of probiotics against depressive symptoms based on the subgroup of studies that recruited participants with neurodevelopmental diagnoses, benefits of certain probiotics in the treatment of specific neurodevelopmental disorders cannot be ruled out. Further large-scale clinical investigations are required to explore the therapeutic potentials of probiotics against different neurodevelopmental disorders.

Apart from the possibility of a differential therapeutic impact of probiotics on various neurodevelopmental disorders, a previous meta-analysis on adults highlighted significant benefits of probiotics in the treatment of depressive symptoms only in those with a diagnosis of anxiety or depressive disorders [5]. Although three out of our five included studies focused on those with a diagnosis of neurodevelopmental disorders [19, 20, 22], none of their participants had a diagnosis of depression or anxiety. While the participants of one study showed minimal depressive symptoms (e.g., mean baseline CDI between 4.1 to 5) [20], the baseline mean CDI score was even higher in the control group (i.e., 12) than that in the probiotics group (i.e., 7) in another trial [22]. On the other hand, the only study that supported the therapeutic effects of probiotics over the controls on the symptoms of depression recruited children and adolescents with a borderline anxiety/depression in both groups based on their CBCL score (i.e., around 64) [19] which was close to the criteria for diagnosing borderline anxiety/depression (i.e., T-score of 65–70) [25]. Therefore, our finding of no significant therapeutic benefit associated with probiotics compared with the controls may be partly attributable to a relatively low depressive symptom severity of the participants. The lack of available study focusing on children and adolescents with a diagnosis of depression warrants further large-scale clinical investigations to elucidate the therapeutic effects of probiotics in these populations.

The results of our secondary outcome showed fair acceptability regarding the use of probiotics among children and adolescents. However, information about the tolerability of probiotics was insufficient for analysis. In addition, we were unable to conduct subgroup analysis targeting the influence of age, duration of treatment, and number of probiotic strains on the therapeutic benefit due to the limited number of available studies.

### Limitations

Several limitations associated with this study need to be considered for correct interpretation of the derived evidence. First, the current investigation was considered a pilot study due to the limited number of available studies with the inclusion of only five trials with 692 participants. Second, the level of evidence of our findings was downgraded to very low because of the high level of heterogeneity across the included studies. Third, limited information about potential confounders including age, treatment duration, number of probiotic strains, as well as factors that may affect treatment outcomes such as dietary habits and consumption of other nutritional supplements precluded our elucidation of their influences. Finally, although previous reports suggested potential treatment benefits of probiotics in those with a neurodevelopmental diagnosis (e.g., ASD) [6] as well as those with more severe depressive symptoms [5], we were unable to reach significant conclusions due to the dearth of studies focusing on children or adolescents with specific neurodevelopmental disorders or targeting those with diagnoses of anxiety or depression. Further large-scale investigations are required to address these issues.

## Conclusions

The current pilot study showed no significant efficacy of probiotics in relieving depressive symptoms compared with controls in children and adolescents diagnosed with neurodevelopmental disorders or in the general population. Nevertheless, given the high level of heterogeneity across the included studies attributable to the recruitment of individuals with different neurodevelopmental disorders and those without such diagnoses, a variation in the severity of depressive symptoms, as well as a lack of studies focusing on those with diagnoses of anxiety or depression, our results could not rule out the therapeutic potential of probiotics against depressive symptoms in these populations. This pilot study encourages further large-scale clinical investigations into the benefits of probiotics among children and adolescents diagnosed with neurodevelopmental disorders or depression.

## Supplementary Information

Supplementary Material 1: eTable 1. Applied keywords and the search results from each database. eTable 2. Reasons for study exclusion. eTable 3. Grading of Recommendations Assessments, Development and Evaluation (GRADE) assessment of the strength of evidence for standard weighted meta-analysis. eFigure 1. Funnel plot – depression. eFigure 2. Odds ratio of dropout rate in Probiotic group compared with control. eFigure 3. Funnel plot – dropout rate.

## Data Availability

The datasets used and/or analyzed during the current study are available from the corresponding author on reasonable request.
