# Supplementary material for: Therapeutic effects of probiotics on symptoms of depression in children and adolescents: a systematic review and meta-analysis
Source: Ital J Pediatr. 2024 Nov 7;50:239. doi: 10.1186/s13052-024-01807-6 (PMC11545225; doi:10.1186/s13052-024-01807-6)

eTable 1. Applied keywords and the search results from each database

Database	Keywords	Filter	Date (yyyy/mm/dd)	Result
PubMed	(probiotics or psychobiotic or probio) and (depression or depressive symptoms or mood)	RCT	2024/05/02	346
Embase	(probiotics or psychobiotic or probio) and (depression or depressive symptoms or mood)	RCT	2024/05/02	237
Cochrane CENTRAL	(probiotics or psychobiotic or probio) and (depression or depressive symptoms or mood)	Trials	2024/05/02	538
ScienceDirect	probiotics and (depression or depressive symptoms or mood)	Research articles	2024/05/02	349
Clinicaltrials.gov	Depression and probiotics	Nil	2024/05/02	49

Abbreviations: NA, not applied; RCT, randomized controlled trial

eTable 2 Reasons for study exclusion

Reason	Number of excluded studies	References
Not targeted at children or adolescents	7	[1-7]
Not Randomized clinical trials	4	[8-11]
No outcome for symptoms of depression	3	[12-14]
No probiotic use	2	[15, 16]

References

1. Zhu, R., et al., *Psychobiotic Lactobacillus plantarum JYLP-326 relieves anxiety, depression, and insomnia symptoms in test anxious college via modulating the gut microbiota and its metabolism.* Front Immunol, 2023. **14**: p. 1158137.
2. Kim, C.S., et al., *Probiotic Supplementation Improves Cognitive Function and Mood with Changes in Gut Microbiota in Community-Dwelling Older Adults: A Randomized, Double-Blind, Placebo-Controlled, Multicenter Trial.* J Gerontol A Biol Sci Med Sci, 2021. **76**(1): p. 32-40.
3. Schaub, A.C., et al., *Clinical, gut microbial and neural effects of a probiotic add-on therapy in depressed patients: a randomized controlled trial.* Transl Psychiatry, 2022. **12**(1): p. 227.
4. Kazemi, A., et al., *Effect of probiotic and prebiotic vs placebo on psychological outcomes in patients with major depressive disorder: A randomized clinical trial.* Clin Nutr, 2019. **38**(2): p. 522-528.
5. Romijn, A.R., et al., *A double-blind, randomized, placebo-controlled trial of Lactobacillus helveticus and Bifidobacterium longum for the symptoms of depression.* Aust N Z J Psychiatry, 2017. **51**(8): p. 810-821.

6. Akkasheh, G., et al., *Clinical and metabolic response to probiotic administration in patients with major depressive disorder: A randomized, double-blind, placebo-controlled trial.* Nutrition, 2016. **32**(3): p. 315-20.
7. Salleh, R.M., et al., *Effects of Probiotics on Anxiety, Stress, Mood and Fitness of Badminton Players.* Nutrients, 2021. **13**(6).
8. Grimaldi, R., et al., *A prebiotic intervention study in children with autism spectrum disorders (ASDs).* Microbiome, 2018. **6**(1): p. 133.
9. Kang, D.W., et al., *Long-term benefit of Microbiota Transfer Therapy on autism symptoms and gut microbiota.* Sci Rep, 2019. **9**(1): p. 5821.
10. Wang, Y., et al., *Probiotics and fructo-oligosaccharide intervention modulate the microbiota-gut brain axis to improve autism spectrum reducing also the hyper-serotonergic state and the dopamine metabolism disorder.* Pharmacol Res, 2020. **157**: p. 104784.
11. Tsai, W.H., et al., *Regulatory effects of Lactobacillus plantarum-GMNL6 on human skin health by improving skin microbiome.* Int J Med Sci, 2021. **18**(5): p. 1114-1120.
12. Liu, Y.W., et al., *Effects of Lactobacillus plantarum PS128 on Children with Autism Spectrum Disorder in Taiwan: A Randomized, Double-Blind, Placebo-Controlled Trial.* Nutrients, 2019. **11**(4).
13. Jacobs, S.E., et al., *Probiotics, prematurity and neurodevelopment: follow-up of a randomised trial.* BMJ Paediatr Open, 2017. **1**(1): p. e000176.
14. Arnold, L.E., et al., *Probiotics for Gastrointestinal Symptoms and Quality of Life in Autism: A Placebo-Controlled Pilot Trial.* J Child Adolesc Psychopharmacol, 2019. **29**(9): p. 659-669.
15. Shadid, R., et al., *Effects of galactooligosaccharide and long-chain fructooligosaccharide supplementation during pregnancy on maternal and neonatal microbiota and immunity--a randomized, double-blind, placebo-controlled study.* Am J Clin Nutr, 2007. **86**(5): p. 1426-37.
16. Kok, C.R., et al., *Stool microbiome, pH and short/branched chain fatty acids in infants receiving extensively hydrolyzed formula, amino acid formula, or human milk through two months of age.* BMC Microbiol, 2020. **20**(1): p. 337.

eTable 3. Grading of Recommendations Assessments, Development and Evaluation (GRADE) assessment of the strength of evidence for standard weighted meta-analysis

Outcome	Design	Risk of bias	Indirectness	Inconsistency	Imprecision	Publication bias	Grade Quality
Depression	RCT x 5	No Serious	No indirectness	Serious inconsistency	Serious imprecision	Undetected	⊕OOO[3,4*]
Numbers of dropouts	RCT x 5	No Serious	No indirectness	No serious inconsistency	Serious imprecision	Undetected	⊕⊕OO[4*]

*Risk of bias was estimated using Cochrane risk of bias, studies were classified as having low risk of bias if none of the domains above was rated as high risk of bias and three or less were rated as unclear risk; moderate if one was rated as high risk of bias or none was rated as high risk of bias but four or more were rated as unclear risk, and all other cases were assumed to pertain to high risk of bias.

Down-graded due to: [1] risk of bias, [2] indirectness, [3.] Inconsistency, [4.] Imprecision, [5.] publication bias

[4*] Down-graded two levels due to very low sample sizes

Abbreviation: ASD autism spectrum disorder

GRADE Working Group grades of evidence:

-**High certainty**: We are very confident that the true effect lies close to that of the estimate of the effect

-**Moderate certainty**: We are moderately confident in the effect estimate: The true effect is likely to be close to the estimate of the effect, but there is a possibility that it is substantially different.

-**Low certainty**: Our confidence in the effect estimate is limited: The true effect may be substantially different from the estimate of the effect.

-**Very low certainty**: We have very little confidence in the effect estimate: The true effect is likely to be substantially different from the estimate of effect.

eFigure 1. Funnel plot – depression

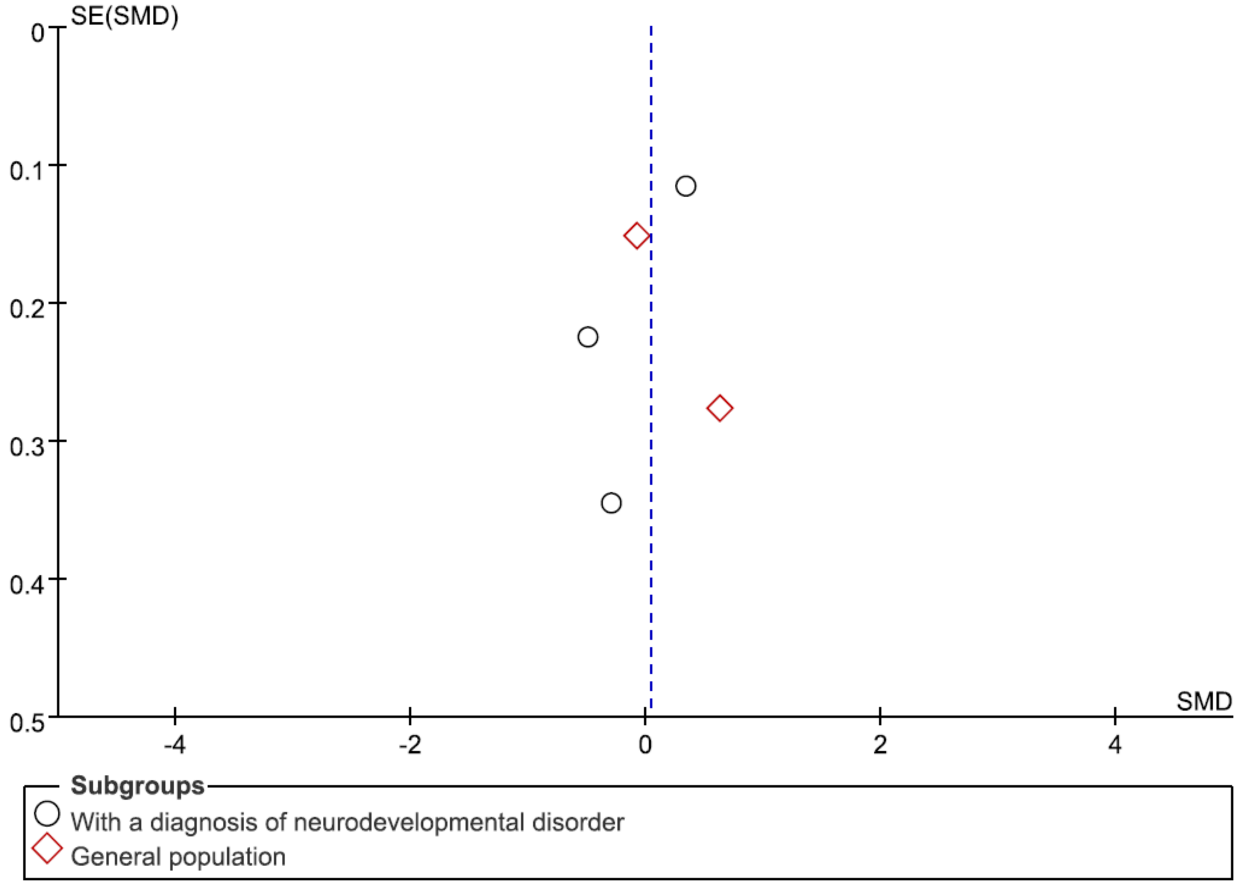

eFigure 2. Odds ratio of dropout rate in Probiotic group compared with control

Study or Subgroup	Probiotics Events	Probiotics Total	Placebo Events	Placebo Total	Weight	Odds Ratio M-H, Fixed, 95% CI	Year
Slykerman (2018)	26	341	21	171	40.3%	0.59 [0.32, 1.08]	2018
Sepehrmanesh (2021)	3	20	3	20	4.0%	1.00 [0.18, 5.67]	2021
Wu (2021)	0	28	0	29		Not estimable	2021
Rianda (2022)	129	244	56	126	54.3%	1.40 [0.91, 2.16]	2022
Liu (2023)	2	41	1	41	1.5%	2.05 [0.18, 23.55]	2023
**Total (95% CI)**		**646**		**358**	**100.0%**	**1.07 [0.76, 1.50]**	
Total events	160		81				

Heterogeneity: Chi² = 5.49, df = 3 (P = 0.14); I² = 45%

Test for overall effect: Z = 0.38 (P = 0.70)

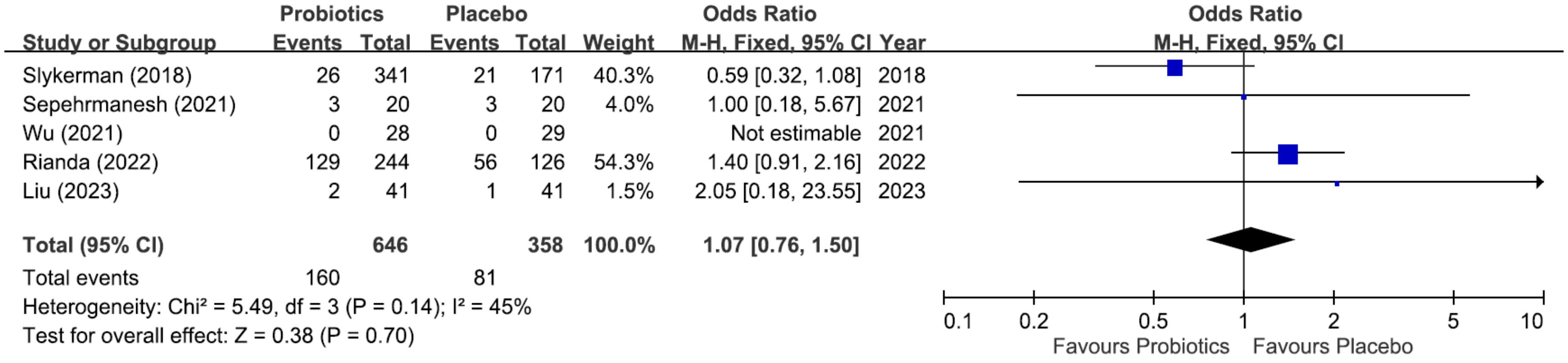

eFigure 3. Funnel plot – dropout rate

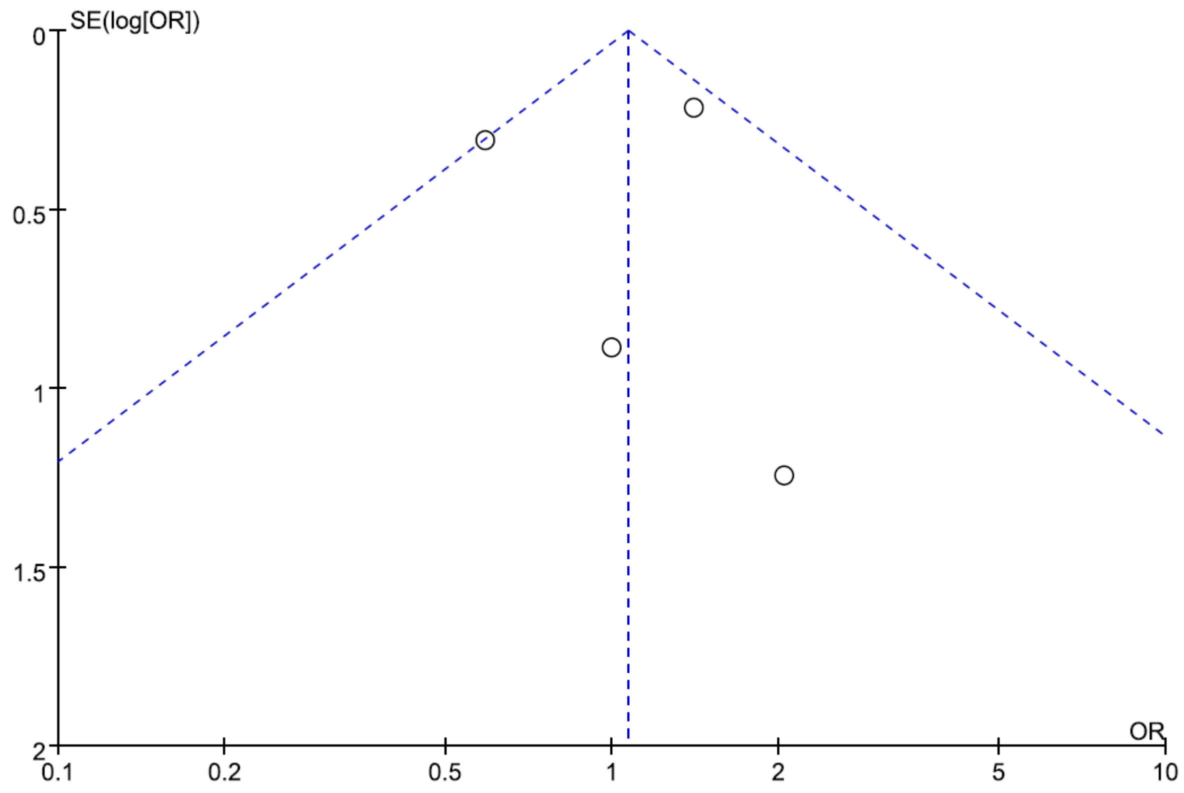

Supplement: Supplementary file 1 — Supplementary Material 1: eTable 1. Applied keywords and the search results from each database. eTable 2. Reasons for study exclusion. eTable 3. Grading of Recommendations Assessments, Development and Evaluation (GRADE) assessment of the strength of evidence for standard weighted meta-analysis. eFigure 1. Funnel plot – depression. eFigure 2. Odds ratio of dropout rate in Probiotic group compared with control. eFigure 3. Funnel plot – dropout rate. [file 13052_2024_1807_MOESM1_ESM.pdf]
